# Supplementary material for: Flexoelectric domain walls enable charge separation and transport in cubic perovskites
Source: Nat Commun. 2026 Feb 16;17:946. doi: 10.1038/s41467-026-68660-5 (PMC12909952; doi:10.1038/s41467-026-68660-5)
Supplement: Supplementary file 1 — Supplementary Information [file 41467_2026_68660_MOESM1_ESM.pdf]

Supplementary Information

Flexoelectric domain walls enable charge separation and transport in cubic perovskites

Dmytro Rak, Dusan Lorenc, Daniel M. Balazs, Ayan A. Zhumekenov, Osman M. Bakr, Zhanybek Alpichshev*

*Corresponding author. E-mail: alpishev@ist.ac.at

Contents

Supplementary Note 1. Temperature dependence of birefringence 2
Supplementary Note 2. Electrochemical staining of domain walls 3
Supplementary Note 3. Photochromic effect 7
Supplementary Note 4. Laue X-ray backscattering study 8
Supplementary Note 5. Optoelectronic measurements 10
Supplementary Note 6. Reconstruction of photocurrent transients 13
Supplementary Note 7. Bulk photovoltaic effect in $MAPbI_3$ 15
Supplementary Note 8. Displacement nature of photocurrent in $MAPbBr_3$ 16
Supplementary Note 9. Fitting the photocurrent decays to different models 17
Supplementary Note 10. Estimation of the characteristic charge density n 17
Supplementary References 20

Supplementary Note 1. Temperature dependence of birefringence

To measure the temperature dependence of birefringence, a small portion of the main beam from an ultrafast laser amplifier (Light Conversion PHAROS HP) was split off, as shown in the schematic in Supplementary Fig. 1. The beam is passed through a Glan-Taylor polarizing prism and a half-wave plate before entering an optical cryostat (Oxford Instruments Optistat CF-V) that holds the sample. The change in the polarization state of the beam after passing through the sample is evaluated by extracting the respective Stokes parameters, following the standard protocol that involves passing the beam through a set of combinations of waveplates and polarizers as described in the literature [1].

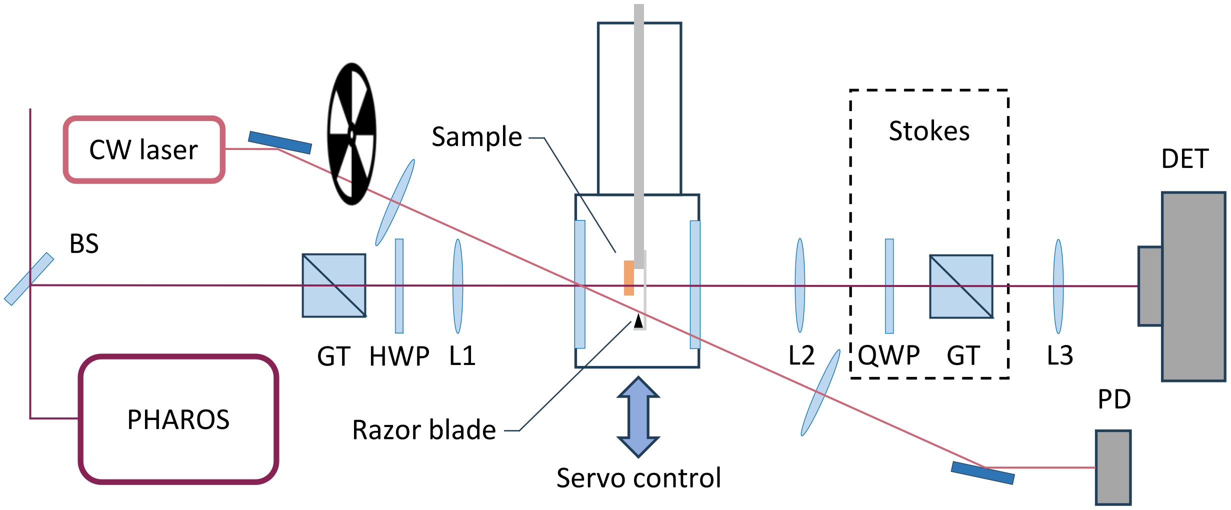

Supplementary Fig. 1. Schematic of experimental setup for temperature-dependent birefringence measurements. CW laser – 635 nm laser diode module; BS – beamsplitter; GT – Glan-Taylor polarizer (Thorlabs GT10); HWP – half-wave plate; QWP – quarter-wave plate; L – lenses; DET – amplified photodetector (Thorlabs PDA100A2); PD – photodiode (Hamamatsu).

In the course of temperature-dependent measurements on a spatially heterogeneous sample, it is of paramount importance to make sure that the probed region remains the same throughout the experiment. The geometry of the experiment (the sample attached to a ~20 cm-long copper cold finger) suggests that the sample shifts by approximately one micron per every degree Kelvin due to thermal contraction of the cryostat components. If this effect is not considered, it will render the data measured at different temperatures incomparable. In order to compensate for sample displacement due to thermal expansion/contraction during large temperature sweeps (>100 K) we have augmented our experimental setup with an active closed-loop control mechanism. To this end, we have mounted a razor blade on the sample holder in close proximity to the sample, on the edge of which a beam from an auxiliary CW laser diode module (λ = 635 nm) was focused (Supplementary Fig. 1). The partially cut 635 nm laser beam was collected at the back window of the cryostat and refocused onto a photodetector. The intensity of the beam was measured by a lock-in amplifier synchronized to an optical chopper modulating the incoming intensity of the 635 nm

laser. The lock-in output was digitized and sent to a PID control system whose output signal operated a servo stage controlling the vertical position of the cryostat. We achieved position stability of the sample, compensating for sample displacements due to thermal expansion with sub-micron precision.

Birefringence values were extracted from the Stokes parameters for each temperature, and the cumulative critical birefringence associated with the cubic-to-tetragonal phase transition Δn_{t} was calculated from the total cumulative birefringence Δn_{c} as $\Delta n_{\mathrm{t}} = \Delta n_{\mathrm{c}} - \Delta n_{\mathrm{f}}$, where Δn_{f} is the "high-temperature" ferroelastic birefringence. The corresponding cumulative retardation $\Delta\Theta$ was then calculated as

$$\Delta\Theta = \frac{2\pi \Delta n_{\mathrm{t}} L}{\lambda}, \tag{1.1}$$

where L is the thickness of the sample, and λ is the wavelength of the probe beam. To extract the value of the critical exponent β, the cumulative retardation $\Delta\Theta$ was fitted to a power law $\Delta\Theta \propto (T_{\mathrm{c}} - T)^{\alpha}$, where $\alpha = 2\beta$. The factor of 2 in the exponent reflects scaling of the birefringence Δn_{t} as the square of the rotation angle of the $PbBr_6$ octahedra ϕ acting as the order parameter: $\Delta n_{\mathrm{t}} \propto \phi$. The obtained value of the critical exponent is $\beta = 0.287$, close to the reported values of 0.19 and 0.25 obtained from diffraction studies of $MAPbBr_3$ and $MAPbI_3$, respectively [2, 3]. The phase transition was observed at the nominal temperature T_{c} = 231.2 K, which is slightly lower than most of the reported values. Several factors can account for the discrepancy with the literature value of T_{c}, including the separation between the sample and the temperature sensor, the low thermal conductivity of the $MAPbBr_3$ combined with constant local heating of the sample by the laser beam, and rather large variability in the reported values of the phase transition temperature [4].

Supplementary Note 2. Electrochemical staining of domain walls

For domain wall visualization in $MAPbBr_3$, silver ions are electrophoretically injected into the sample. The injected ions diffuse preferentially along the domain walls and are electrochemically reduced to metallic silver, rendering the domain walls visible. Several factors enable this approach. First, the redox potential of the Ag^{+1}/Ag^{0} couple in silver bromide is only 0.071 V [5]. As a result, both the oxidation of metallic silver to Ag^{+1} and the reduction of Ag^{+1} to metallic silver in the presence of Br^{-1} have extremely low activation energy and can be catalyzed by a range of stimuli, including electrical current. Second, the electrophoretic transport of ions into the bulk of the crystal is enhanced by the exceptionally high mobility of Ag^{+} ions [6] and by increased ionic diffusion along domain walls [7, 8]. Finally, the accumulation of charge carriers at domain boundaries increases the probability of encounters between conductive electrons and silver ions, leading to their reduction to metallic silver at the precise locations of the domain walls.

Two complementary imaging methods—bright-field and confocal microscopy—were used to acquire microscopic images of the samples, allowing visualization of the three-dimensional domain structure. In bright-field mode, all transmitted light is collected, producing images in which darker regions correspond to areas that absorb or scatter light, providing a general view of

the domain wall morphology. In confocal mode, a pinhole is placed in front of the detector to block out-of-focus light, which enhances image resolution and contrast. This approach allows the acquisition of sharp, thin optical slices at precise depths within the sample. By collecting a series of such slices, the internal structure of the sample could be reconstructed in three dimensions, revealing features that would be obscured in conventional bright-field imaging.

Depending on the applied voltage and treatment duration, silver structures of different morphologies form within single-crystal samples. Supplementary Fig. 2b shows a high-resolution image (0.69 μm per pixel) of silver dendrites in the $MAPbBr_3$ sample, revealing domains as small as 5×5 μm^2. The small domain sizes help explain the X-ray study results, including those in our work (Supplementary Note 4), which identified the high-temperature phase of $MAPbBr_3$ as cubic $Pm\bar{3}m$. Given that most X-ray sources have spot sizes spanning several hundred micrometers, the structure appears cubic on average despite consisting of multiple domains with different strain orientations.

Microscopic images of electrochemically stained samples reveal domain boundaries oriented at 45° and 90° to the crystal axes, suggesting the presence of 90° and 180° domain walls. The prevalence of 90° walls may indicate either a higher concentration of such walls or a greater likelihood of silver ion reduction occurring at these walls. Both explanations are plausible: 90° walls were observed in the tetragonal phase of oxide and lead-halide perovskites [4, 9], and the accumulation of silver ions, conductive electrons, or both along 90° walls could increase the probability of silver ion reduction at these specific locations.

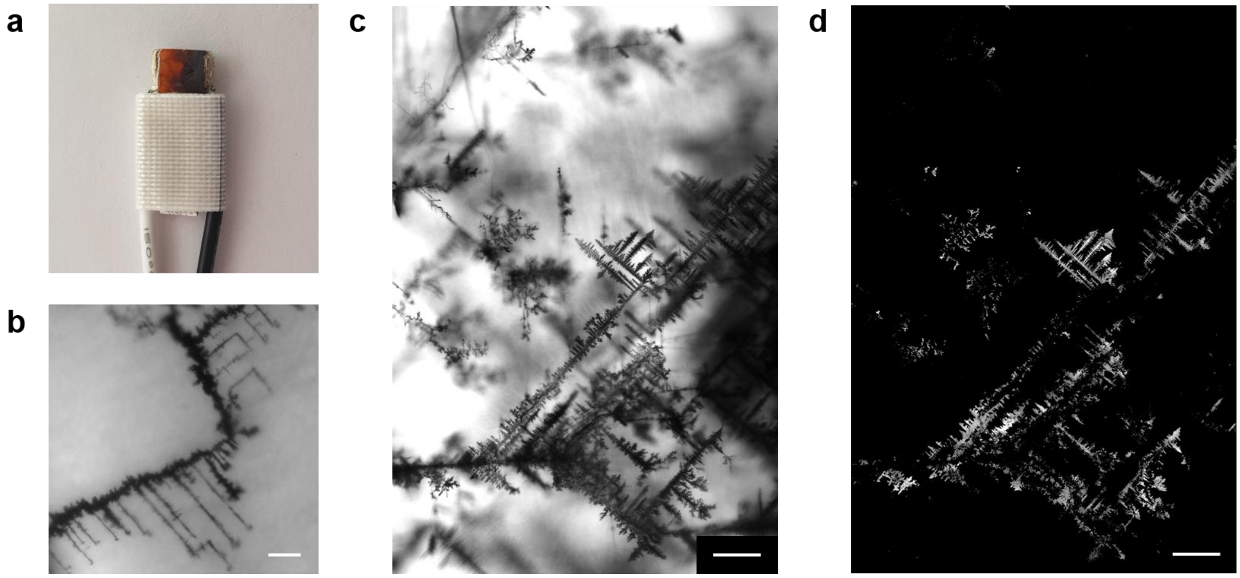

Supplementary Fig. 2. Visualization of the domain structure in $MAPbBr_3$ single crystal samples. a $MAPbBr_3$ sample with attached silver contacts after an electric field of 72 V mm^{-1} was applied for 1 hour. **b** Bright-field image of silver dendrites formed in the $MAPbBr_3$ sample shown in (a). The image shows a single optical slice taken at 147 μm beneath the sample surface. The scale bar is 50 μm. **c, d** Bright-field (c) and confocal (d) images of silver dendrites formed in the $MAPbBr_3$ sample after an electric field of 24 V mm^{-1} was applied for 4 hours. The images show a single optical slice taken 325 μm beneath the sample surface. The scale bars are 200 μm.

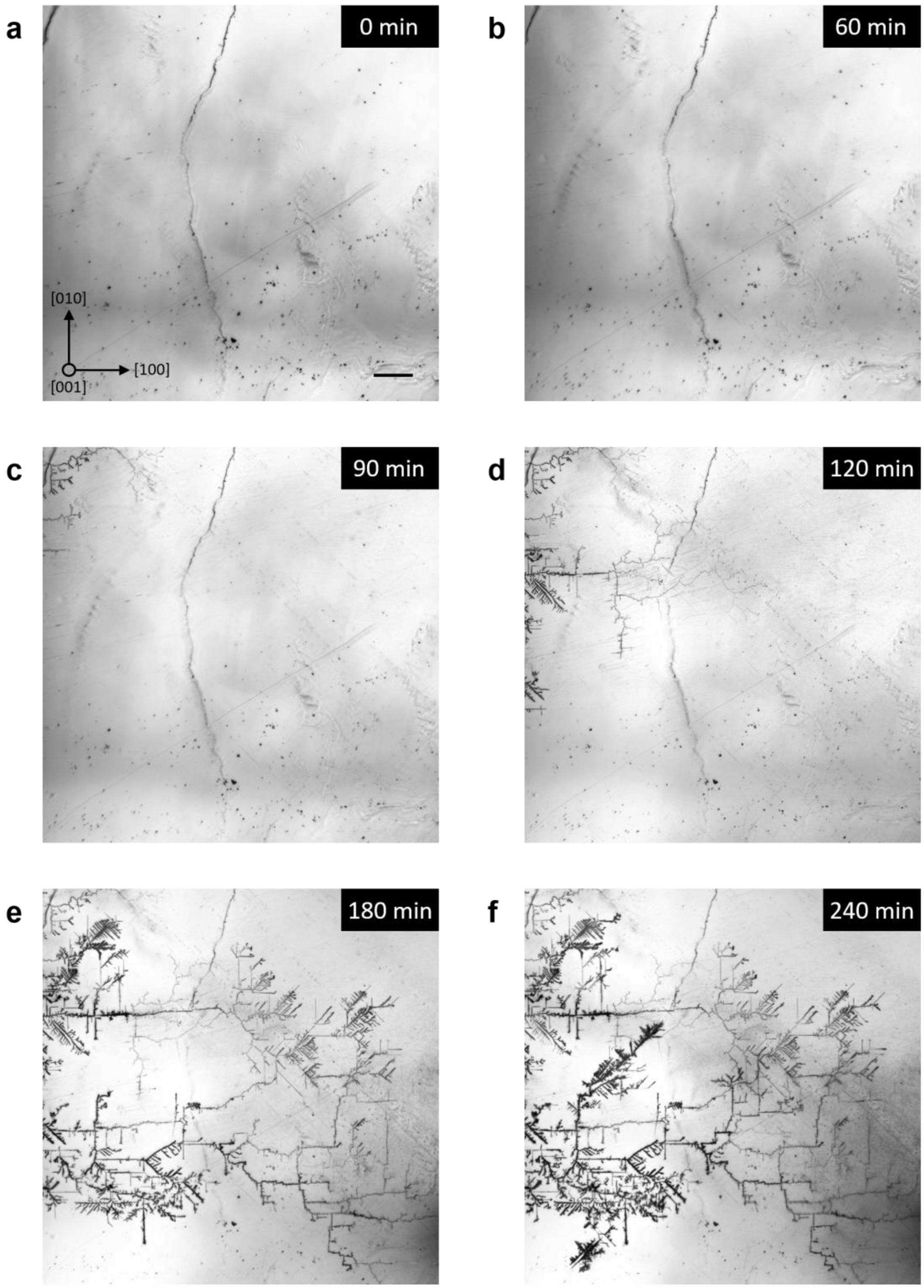

Supplementary Fig. 3. Electrochemical staining of the domain walls in a typical $MAPbBr_3$ single crystal sample as a function of time. Bright-field microscopic images show a pre-selected part of the sample monitored during the electrochemical staining process. The microscopic images were taken immediately after the electric field was removed. The imaged silver structures are located 31 μm beneath the sample surface. The scale bar is 100 μm.

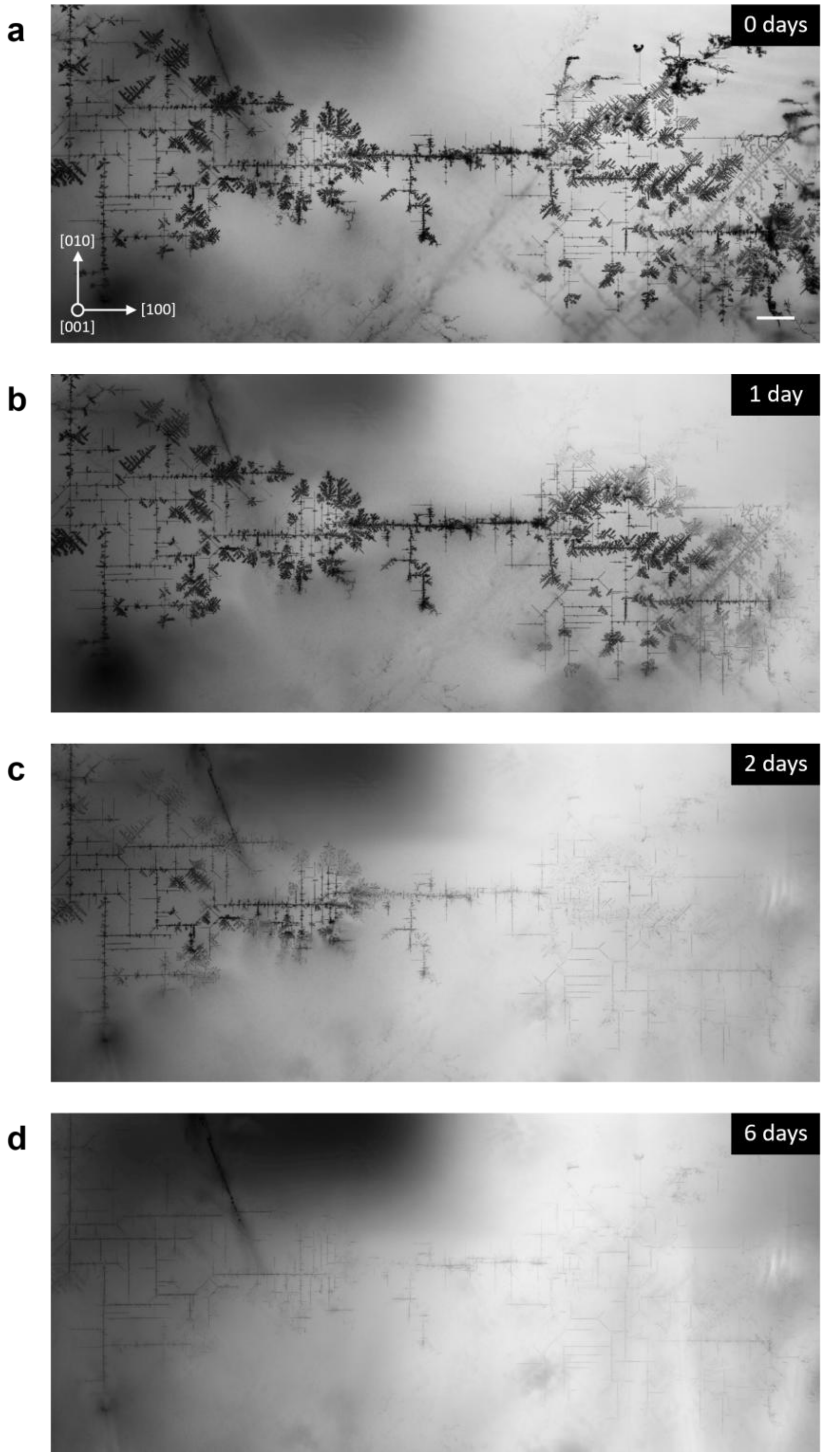

Supplementary Fig. 4. Dissolution of silver dendrites formed in a typical $MAPbBr_3$ single crystal sample. Bright-field microscopic images of silver dendrites formed in the $MAPbBr_3$ monocrystal located 194 µm (c) beneath the sample surface. An electric field of 100 V mm^{-1} was applied for 4 hours. The microscopic images were taken immediately after the electric field was removed (**a**), after 1 day (**b**), after 2 days (**c**), and after 6 days (**d**), respectively. The scale bar is 100 µm.

Supplementary Fig. 3 illustrates the time-dependent electrochemical staining of domain walls in the $MAPbBr_3$ single crystal sample. Since the microscope used for tracing the growth of dendrite structure does not allow for the *in situ* application of voltage inside the microscope, an electric field of 100 V mm^{-1} was applied in successive steps, with 40–60 minute pauses after each step to allow imaging of a pre-selected region of the sample. Microscopic images were captured at intervals corresponding to 0, 30, 60, 90, 120, 180, and 240 minutes of cumulative application of the electric field.

No noticeable changes were observed up to 60 minutes (Supplementary Fig. 3a, b) of cumulative field application. The first silver-stained domain walls appeared after 90 minutes (Supplementary Fig. 3c), and their number increased progressively with longer exposure. The domain wall patterns that became visible after 90, 120, and 180 minutes (Supplementary Fig. 3c-e) remained stable during further application of the electric field, demonstrating that the electric field does not alter the intrinsic domain structure of $MAPbBr_3$ but only reveals pre-existing domain walls through the staining process. No attempt was made to track the evolution of the structures beyond 240 minutes (Supplementary Fig. 3f) due to the high risk that silver dendrites would reach the opposite side of the crystal, connect the electrodes directly, and cause a short circuit, which could destroy the sample and potentially damage the equipment.

The silver structures begin to dissolve once the electric field is removed, indicating their nonequilibrium nature. Therefore, it should be considered that since microscopic imaging is a time-consuming process that can take several hours, structures formed through a step-by-step process may not be identical to those produced by a single-step procedure. Periodic application of an electric field with sufficiently long off-times, allowing the already formed structures to partially dissolve, could have unpredictable effects on the final structures.

Supplementary Fig. 4 illustrates the gradual disappearance of silver structures formed within the $MAPbBr_3$ single crystal. Following electrochemical staining, the sample was stored at room temperature in a nitrogen atmosphere. Over the course of six days, the silver-stained domain walls became barely visible, indicating nearly complete dissolution of metallic silver. Since dissolution is a chemical process, its rate can vary significantly depending on the specific sample and storage temperature.

Supplementary Note 3. Photochromic effect

A photochromic effect was observed on the $MAPbBr_3$ sample doped with silver ions using the procedure described in the Methods section of the main text. An electric field of 6 V mm^{-1} was applied for 4 hours. Neither the formation of the silver structures nor the visible darkening of the sample was observed during this period. Subsequently, the sample was used in optical measurements. A small fraction of the beam (1 mW average power) from an ultrafast laser amplifier (Light Conversion PHAROS HP) with central wavelength λ = 1028 nm, pulse duration τ = 5 ps, and repetition rate of 1.5 kHz was focused on the central part of the sample. The beam waist diameter was w = 40 µm. The transmitted beam was collected and refocused onto the photodiode detector (Thorlabs PDA100A2), which was connected to the lock-in amplifier.

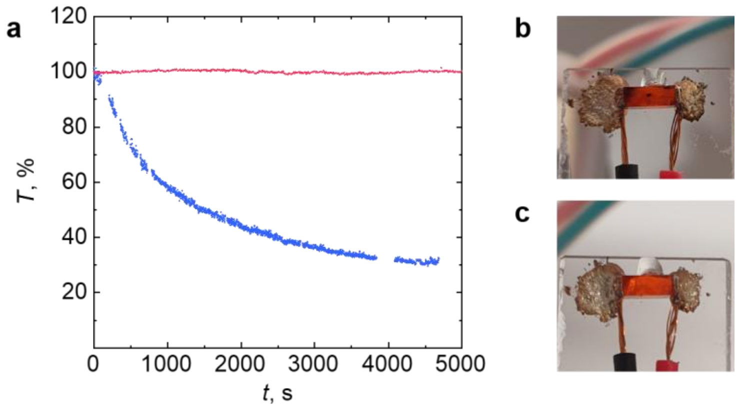

Supplementary Fig. 5. Photochromic effect in silver-doped $MAPbBr_3$ single crystal sample. a Evolution of 1028 nm light transmission through pristine (pink dots) and silver-doped $MAPbBr_3$ samples (blue dots) as a function of time. **b** Photograph of the $MAPbBr_3$ crystal showing a dark spot at the location where the laser beam was focused. **c** Photograph of the sample after it was heated to 60°C for a few minutes.

As soon as the experiment started, a gradual decrease in the transmitted beam intensity was observed (Supplementary Fig. 5a). In just over an hour, the apparent saturation was reached with a resulting optical density of the sample of approximately 0.5. During the measurement, a visible dark cone formed within the bulk of the sample, extending through the entire thickness of the crystal and reaching its opposite side (Supplementary Fig. 5b). The dark cone completely disappeared within minutes after the sample was heated to 60°C (Supplementary Fig. 5c). Such behavior resembles that of silver-halide-sensitized photochromic glasses, where darkening results from the photochemical decomposition of silver halide nanocrystals embedded in the silicate glass matrix [10]. In the latter, the silver ions capture photoelectrons ejected from the halogen ion by incident UV or visible light, resulting in the formation of small aggregates of silver atoms absorbing light. Since the reaction products remain in the reaction volume, the process is completely reversible, and silver nanoclusters dissolve when the glass is heated or left unperturbed in darkness at room temperature. The mechanism of the photochromic effect in $MAPbBr_3$ samples with electrophoretically injected silver thus appears to be very similar in nature to that in silver halide-doped photochromic glasses, although in this case, photoelectrons are produced through a two-photon absorption process. When comparing the saturation optical density of the silver-doped sample to that of the photochromic glasses [10], the silver concentration can be roughly estimated to be 0.5% by mass. However, other factors, such as the fading rate, might influence the steady-state optical density. The photochromic effect was not observed in undoped $MAPbBr_3$ under the same experimental conditions.

Supplementary Note 4. Laue X-ray backscattering study

Laue X-ray backscattering measurements were performed on the same $MAPbBr_3$ sample that was used for birefringence measurements shown in Fig. 1d and photocurrent measurements presented

in Fig. 3 and Fig. 4. The sample was cleaved along the (001) crystal plane approximately halfway through the thickness of the crystal to expose a fresh surface from within the bulk and mounted onto a sample holder. Laue backscattering images were collected on a Photonic Science Laue crystal orientation system. The sample was placed under a white X-ray beam (5-50 keV) with a beam footprint of approximately 400 μm, and the backscattered photons were detected using a 2D CCD-scintillator screen. The sample-to-detector distance was set to 47.6 mm. The pixel size after binning was 180 μm. Eight images with a 0.5 mm interval along the long sample axis were collected (Supplementary Fig. 6a). The orientation was confirmed by fitting the measured data to the previously reported $Pm\bar{3}m$ crystal structure (COD entry 1545320); the (100) lattice planes were found to be approximately parallel to the crystal facets. Positions of the strongest 33 peaks were extracted from all images by fitting a "round" 2D Gaussian peak using an in-house-written MATLAB code. Peaks that could not be reliably fit for all 8 images were excluded. A peak position uncertainty of 1 pixel was assumed and marked on the plots (Supplementary Fig. 6b); all positions fell within this range. The peak intensity uncertainty was set to be 1 count.

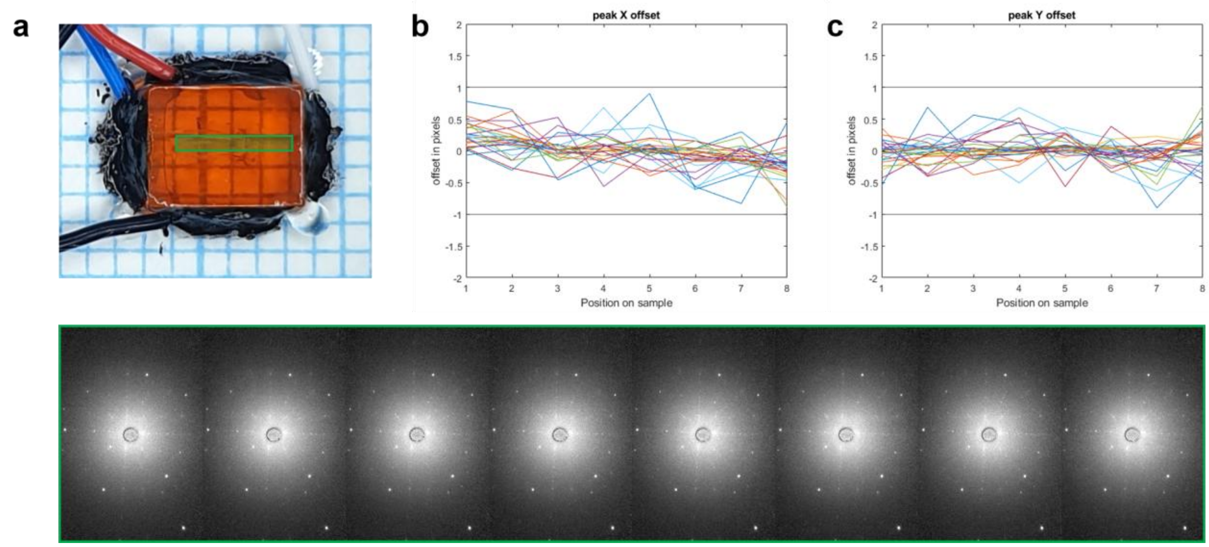

Supplementary Fig. 6. Laue X-ray backscattering. a Photograph of the $MAPbBr_3$ sample with attached carbon epoxy contacts. The green rectangle shows the part of the sample where the Laue backscattering images shown below were taken. **b** X-coordinate offsets for the 33 strongest reflections plotted as a function of the beam position on the sample. **c** Y-coordinate offsets for the 33 strongest reflections plotted as a function of the beam position on the sample.

No qualitative differences were observed between the patterns, indicating that all measured spots belong to a single domain. This contradicts the significant variations in birefringence and photocurrent observed across different regions of the sample (Figs. 1d and 3c). This discrepancy can be attributed to the fact that the sizes of the domains revealed through electrochemical staining of the domain walls are much smaller than the spot size of the X-ray source. Consequently, structural differences between individual domains average out, making the crystal structure appear cubic. The X-ray findings rule out a "hard" structural phase with well-defined lattice periods as a

possible explanation for the observed optical anisotropy of $MAPbBr_3$, supporting instead the ferroelastic nature of the high-temperature phase.

Supplementary Note 5. Optoelectronic measurements

The bulk photovoltaic effect was measured in two separate experimental arrangements. In the first case, the sample was enclosed in a light-tight box isolating it from the stray light (Supplementary Fig. 7a), and a horizontally polarized pulsed sub-bandgap pump (1028 nm, 0.250 mW, 290 fs, 1.5 kHz repetition rate) was passed inside the box through a bandpass filter (Thorlabs FLH1030-10). In the second case, measurements were performed without the box under a diffuse ambient light level of 0.01 sun (Supplementary Fig. 7b).

The results of the position-dependent measurements were unaffected by scan direction and were consistently observed in repeated experiments. The photocurrent generated at each spot could be reproducibly measured independently, without scanning the entire crystal, as demonstrated by the multiharmonic measurements used to reconstruct photocurrent transients (see Supplementary Note 6). The magnitudes of I_{rep} obtained in these single-spot measurements are in good agreement with those from complete area scans, within the experimental error. For example, the I_{rep} photocurrent at position 1 in Fig. 4a measured for transient reconstruction was 1.55×10^{-11} pA, closely matching the mean value of $1.45 \times 10^{-11} \pm 1.98 \times 10^{-12}$ pA obtained from five independent area scans, demonstrating the reproducibility and consistency of the measurements.

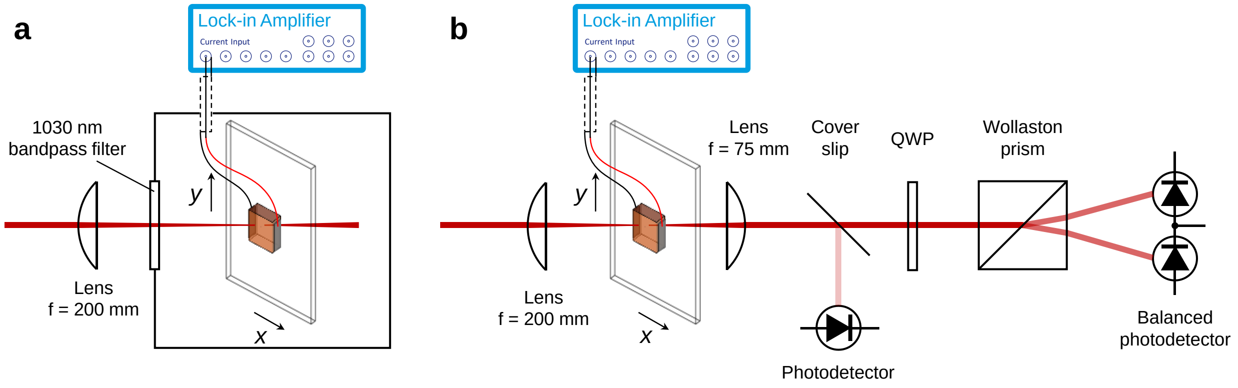

Supplementary Fig. 7. Schematics of two different experimental arrangements for the photocurrent measurements. a $MAPbBr_3$ sample is enclosed in a light-tight box isolating it from the stray light. Only the photocurrent is measured. **b** Measurements are performed in the presence of low-intensity ambient light. Photocurrent, transmission, and birefringence are measured simultaneously. QWP – quarter-wave plate.

Supplementary Fig. 8 shows the results of the position-dependent measurements performed under ambient illumination. The photocurrent was measured without applying the bias voltage, as described in the Methods section of the main text. Ellipticity acquired by a horizontally polarized pump after passing through the sample was resolved using a combination of a quarter-wave plate

and a Wollaston prism followed by an in-house-built balanced photodetector connected to a lock-in amplifier referenced to a laser output. A small fraction of the beam was split with a coverslip glass and guided into an amplified photodetector (Thorlabs PDA100A2) to monitor the transmission. To measure photoluminescence, the quarter-wave plate and Wollaston prism were removed, and the balanced photodetector was replaced with an avalanche photodiode detector (Becker & Hickl APM-400-P-078). The emitted light was focused onto the photodiode, while the residual pump was filtered out using a short-pass filter.

The map of the photocurrent measured horizontally (Supplementary Fig. 8a) resembles the one obtained with the sample placed inside the light-tight box (Fig. 3e). However, the detected photocurrent is significantly higher due to the presence of ambient light, which increases the concentration of free carriers and consequently affects the tunneling rate that determines the magnitude of the photocurrent (see the Discussion section of the main text). The image of the sample constructed from position-dependent measurements of transmitted light (Supplementary Fig. 8b) reveals optical inhomogeneities associated with surface defects resulting from interaction with the glass substrate during crystal growth. The same defects are visible in the area scan of photoluminescence shown in Supplementary Fig. 8d. These defects have no observable effect on the measured photocurrent since the signal primarily originates from the bulk. The reconstructed birefringence map (Supplementary Fig. 8c) corresponds to the transmission image of the sample in a crossed-polarizer setup illuminated by a horizontally polarized 632.8 nm He-Ne laser (Fig. 1c, d). The photoluminescence presented in Supplementary Fig. 8d is uniform across the sample, indicating that the spatial distribution of the photocurrent magnitude does not correlate with the number of generated photocarriers. This further supports the notion that internal electric fields are the source of the observed bulk photovoltaic effect.

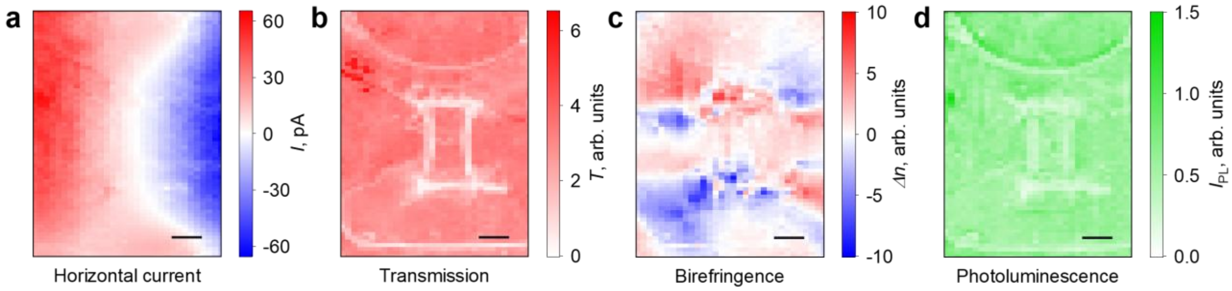

Supplementary Fig. 8. Photocurrent, optical transmission, birefringence, and photoluminescence measurements on the $MAPbBr_3$ sample under low-intensity (0.01 sun) diffuse ambient illumination. a Spatial distribution of the photocurrent I_{rep} measured in the horizontal direction at a pump laser repetition rate of f_{rep} = 1.5 kHz in the presence of low-intensity ambient light. **b** Transmission image of the sample obtained by point-by-point scanning with a 1028 nm pump beam during photocurrent measurements. **c** Spatial distribution of birefringence recorded during photocurrent measurements. **d** Spatial distribution of photoluminescence measured separately on the same sample. The scale bars are 500 µm.

To confirm that the pump beam (1028 nm, 0.250 mW, 290 fs, 1.5 kHz repetition rate) with a 40 µm beam waist used in our photocurrent experiments did not cause nonlinear damage, we

carried out time-dependent measurements of transmission and photoluminescence on a typical $MAPbBr_3$ single crystal sample (Supplementary Fig. 9). No significant changes in transmission or photoluminescence were observed throughout the entire experiment. Since measuring a single harmonic of photocurrent takes only 10 seconds, and approximately 6 minutes are needed to measure the signal at multiple harmonics of the laser repetition rate to reconstruct the pulse shape, it is unlikely that the beam caused any nonlinear damage to the sample during the experiment.

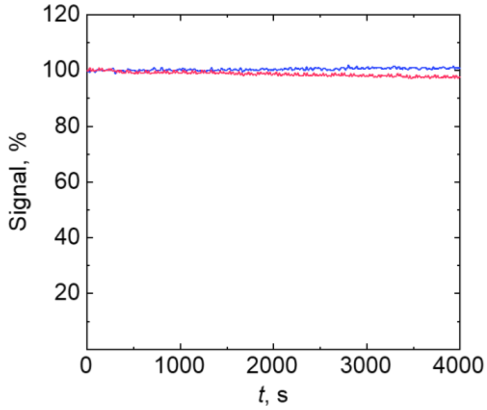

Supplementary Fig. 9. Stability of a typical MAPbBr single crystal sample. Transmission (blue line) of the 1028 nm pump beam and photoluminescence (pink line) excited by the 1028 nm pump beam as a function of time.

Supplementary Fig. 10 shows the power dependence of photoluminescence in a typical $MAPbBr_3$ single crystal sample. At low excitation powers, the photoluminescence intensity follows a power law $I \propto P^2$—a clear signature of a two-photon absorption process. At higher powers, saturation effects reduce the apparent slope.

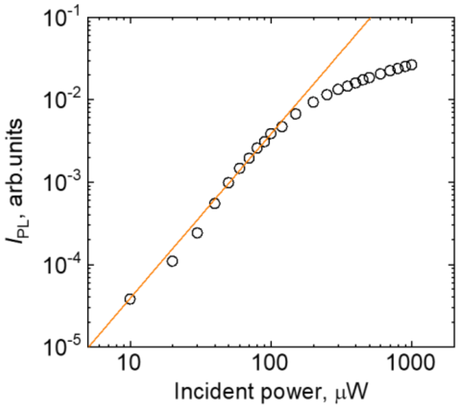

Supplementary Fig. 10. Power-dependent photoluminescence of a typical $MAPbBr_3$ single crystal sample. Photoluminescence excited by the 1028 nm pump beam as a function of incident pump power. The initial slope (first 12 datapoints) was fitted with $f(x) = ax^b$, yielding b = 2.00 with goodness-of-fit R^2 = 0.99.

Supplementary Note 6. Reconstruction of photocurrent transients

To obtain the time-resolved photocurrent transients, the frequency components of the current transients were measured separately at integer multiples of the laser repetition rate using the lock-in amplifier. The time-domain representations of the signal were then reconstructed by inverse Fourier transform of the frequency-domain data (see the Methods section of the main text). Although the lock-in amplifier (Zurich Instruments MFLI) provides a bandwidth of 450 kHz at the 1 µA current input range, only the first n = 102 harmonics were measured, resulting in an effective bandwidth of 153 kHz. The reason for this was to minimize sample exposure to the pump beam and prevent potential damage to the sample. For the area scans used to extract the currents I_0 and the decay rates $1/\tau$ shown in Fig. 4b, c, the number of harmonics was limited to n = 18 because of the extensive measurement times required to acquire the data.

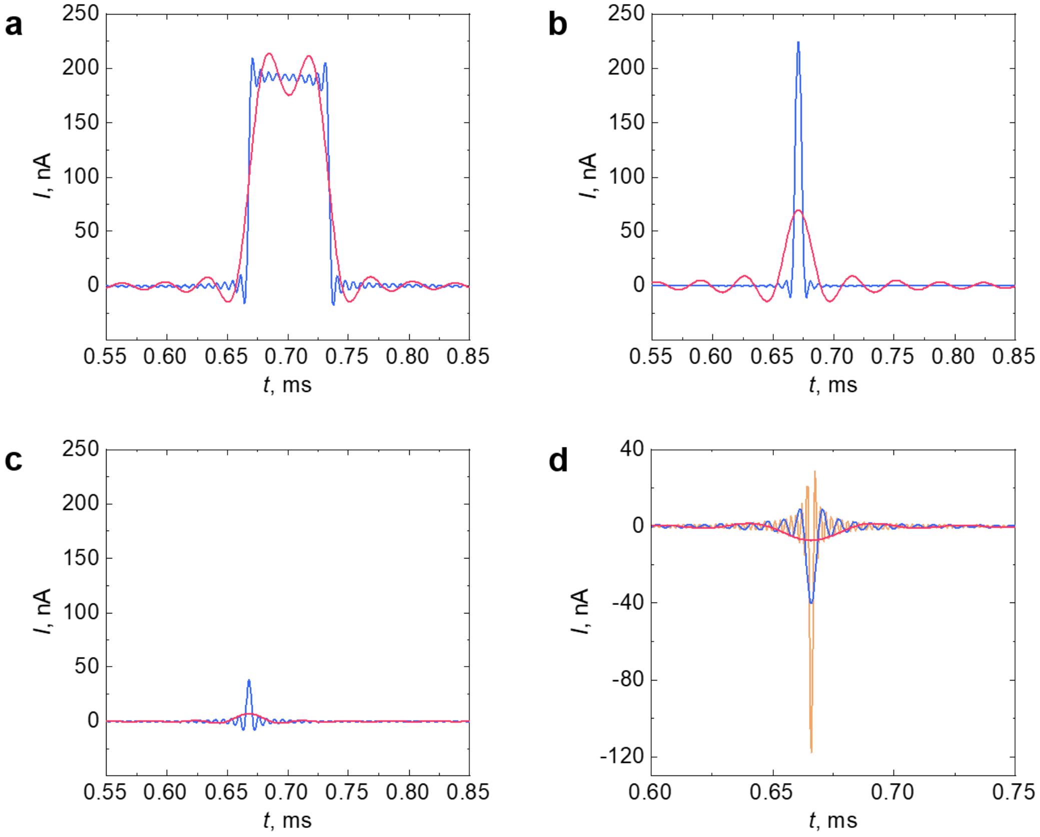

Supplementary Fig. 11. Reconstruction of test current pulses. a-c Positive square pulses at a repetition rate of 1.5 kHz and duty cycles of 10%, 1%, and 0.1%, respectively, reconstructed using the first n = 102 (blue) and n = 18 (pink) harmonics of the repetition frequency. **d** Negative square pulses at a repetition rate of 1.5 kHz and a duty cycle of 0.1% reconstructed using the first n = 300 (orange), n = 102 (blue), and n = 18 (pink) harmonics of the repetition frequency.

The reconstruction procedure was tested on a set of nearly square pulses with known durations and amplitudes at a repetition rate of 1.5 kHz (Supplementary Fig. 11a-c). Positive pulses with

amplitudes of 200 mV and duty cycles of 10%, 1%, and 0.1% were generated using a Rigol DG812 arbitrary waveform generator. The output from the signal generator was connected to the current input of the lock-in amplifier through a 1 MOhm resistor. The corresponding current waveforms were reconstructed from the frequency domain data using a procedure described in the Methods section of the main text. As shown in Supplementary Fig. 11a, the reconstruction of the signal using n = 18 harmonics results in a good reproduction of the square pulses with a 10% duty cycle; however, fine features, such as sharp edges, are lost. Reliable reconstruction of the signals with a 1% duty cycle can be achieved for a number of harmonics n = 102 (Supplementary Fig. 11b). Pulses with a duty cycle of 0.1% (duration under 1 μs) cannot be accurately reconstructed if the number of harmonics is limited to n = 18 or n = 102 (Supplementary Fig. 11c). Loss of high-frequency components of the signal leads to the loss of sharp features in the reconstructed current transients. For this reason, the current spikes observed during polarization buildup, caused by the separation and entrapment of charge carriers at the domain walls in regime I (Fig. 3e, the Discussion section of the main text), are significantly reduced or entirely absent in most of the reconstructed current transients presented in this work. A faithful reconstruction of the features with characteristic times below 1 μs would require a significantly higher number of harmonics. For instance, reconstructing square pulses with a duty cycle of 0.1% (Supplementary Fig. 11d) yields unsatisfactory results, even when the number of harmonics is increased to n = 300, reaching the bandwidth limit of our lock-in amplifier (450 kHz) for the respective current range.

Supplementary Fig. 12 compares photocurrent waveforms reconstructed using the first n = 102 and n = 18 harmonics of the pump laser repetition frequency. It is evident that slow current decays are reproduced with adequate precision even when the number of harmonics is limited to n = 18. To obtain the currents I_0 and decay rates $1/\tau$ shown in Fig. 4b, c, the current decays reconstructed using the first n = 18 harmonics were fitted to an exponential function. For the plot in Fig. 4d, which illustrates the correlation between currents I_0 and decay rates $1/\tau$, only values from fits with a goodness-of-fit of $R^2 \geq 0.9$ were used. The rest of the data were excluded from the analysis.

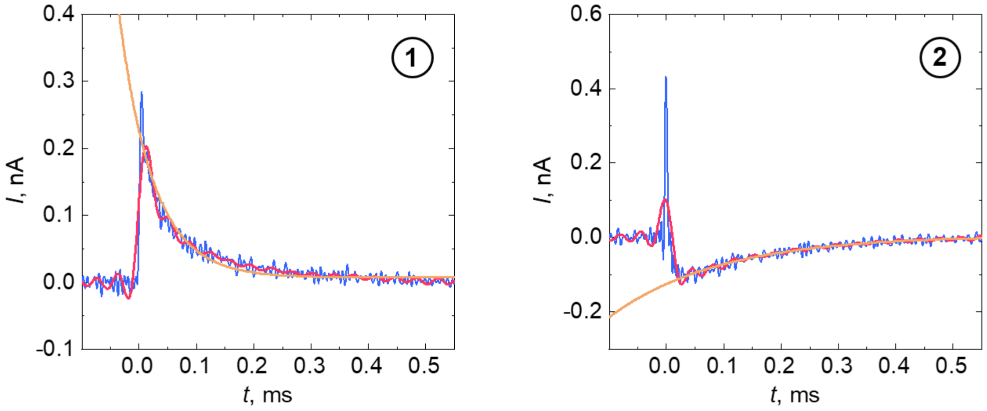

Supplementary Fig. 12. Reconstruction of the photocurrent transients. Photocurrent transients reconstructed using the first n = 102 (blue) and n = 18 (pink) harmonics of the pump laser repetition frequency $f_{\mathrm{rep}} = 1.5\ kHz$. The measurements were performed in the horizontal direction on a sample isolated from the stray light at the locations indicated in Fig. 4b. The orange curves are exponential fits to the data.

Supplementary Fig. 13a shows photocurrent transients reconstructed using $n = 102$ harmonics measured on a sample isolated from stray light (blue curves) and under diffuse ambient illumination of 0.01 sun (pink curves) at different regions of the sample. The measurement positions are indicated on the horizontal I_{rep} current map (Supplementary Fig. 13b), which corresponds to the dataset presented in Fig. 3e.

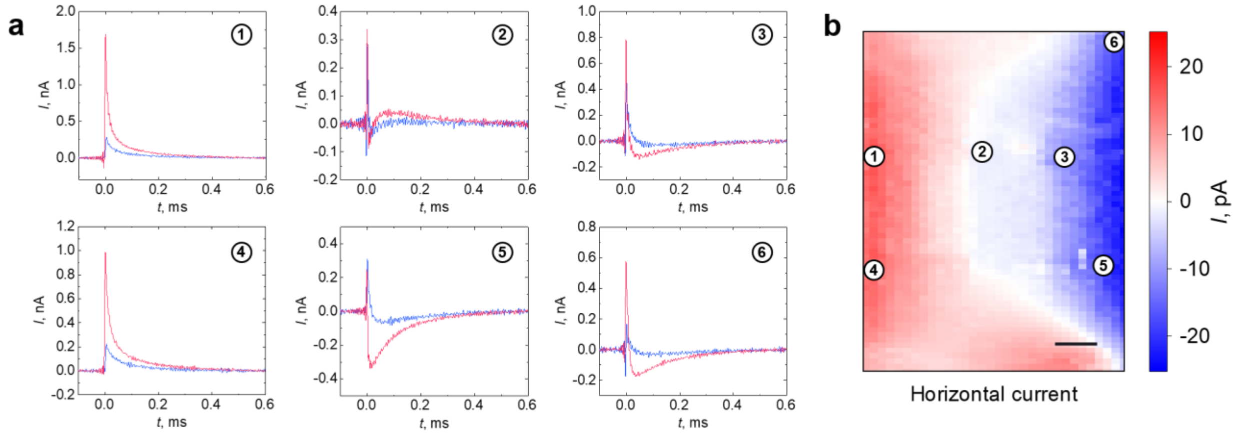

Supplementary Fig. 13. Dependence of photocurrent on ambient illumination. a Photocurrent transients at the marked positions in (a), reconstructed from the first $n = 102$ harmonics of the laser repetition frequency. Different colors represent measurements performed on a sample isolated from stray light (blue) and under diffuse ambient illumination of 0.01 sun (pink), respectively. **b** Spatial distribution of the photocurrent I_{rep} measured in the horizontal direction obtained by local excitation of the $MAPbBr_3$ single crystal sample. Numbers indicate the positions where multiharmonic measurements were taken to reconstruct photocurrent transients. The scale bar is 500 µm.

Supplementary Note 7. Bulk photovoltaic effect in $MAPbI_3$

The $MAPBI_3$ crystals were grown using the following procedure. A 1.2 M solution of CH_3NH_3I/PbI_2 in γ-butyrolactone (GBL) was prepared, filtered through a 0.45 µm-pore-size PTFE filter, and the vial containing 0.5-1 ml of the solution was placed on a hot plate at 70°C. Then the solution was gradually heated to ~100°C and kept at this temperature until the formation of $CH_3NH_3PbI_3$ crystals. The crystals can be grown into larger sizes by elevating the temperature further. The crystals were collected and cleaned using a Kimwipe paper. The CH_3NH_3I (>99.99%) was purchased from GreatCell Solar Ltd. (formerly Dyesol), GBL (≥99%) was purchased from Sigma Aldrich, and PbI_2 (>99.0%) was purchased from Tokyo Chemical Industry Co. Ltd. All chemicals were used as received.

The measurements of zero-bias photocurrent in a single crystal sample of $MAPbI_3$ were performed under the same experimental conditions as measurements in $MAPbBr_3$. The carbon epoxy contacts were attached to the opposite sides of the sample (Supplementary Fig. 14). The sample was then enclosed in a light-tight box isolating it from the stray light, and a horizontally polarized pulsed sub-bandgap pump (1028 nm, 0.100 mW, 290 fs, 1.5 kHz repetition rate) was passed inside the box through a bandpass filter (Supplementary Fig. 7a). The photocurrent was measured using lock-

in amplifier without applied bias. The time-resolved photocurrent transients were reconstructed following the procedure described in the Methods section of the main text.

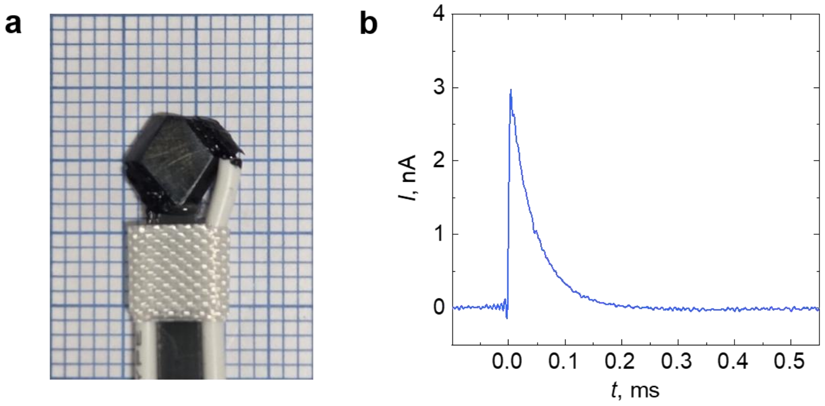

Supplementary Fig. 14. Bulk photovoltaic effect in $MAPbI_3$. a Photograph of the $MAPbI_3$ sample with attached carbon epoxy contacts. **b** Photocurrent transient reconstructed using the first n = 102 harmonics of the repetition frequency.

Supplementary Note 8. Displacement nature of photocurrent in $MAPbBr_3$

Lock-in detection with a finite harmonic cutoff, followed by truncated Fourier reconstruction, leads to a loss of high-frequency signal components—such as the fast spike associated with charge separation in regime I (Fig. 3g, Discussion section of the main text). As a result, integration of the reconstructed current transients does not yield the true transferred charge. Instead, it reflects only the contribution from the bandwidth-limited signal. The apparent average photocurrent obtained by integrating transients reconstructed from the first n harmonics of the pump laser repetition frequency can therefore be referred to as the band-limited photocurrent.

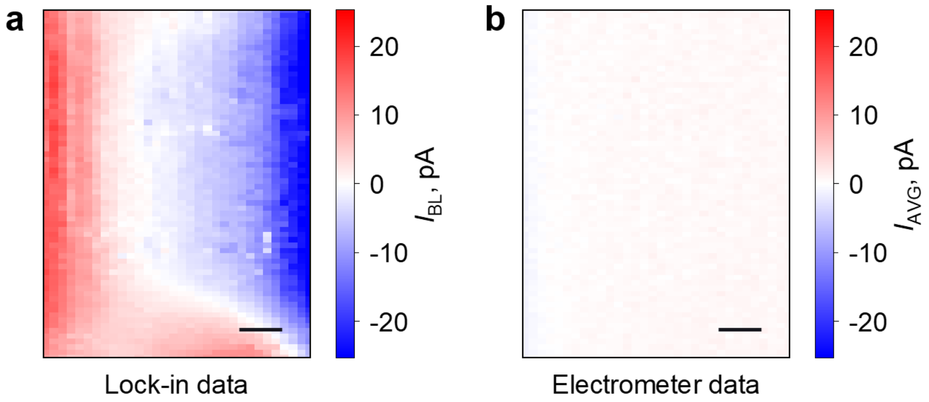

Supplementary Fig. 15. Spatial distributions of band-limited and average photocurrent. a Spatial distribution of band-limited horizontal photocurrent obtained from truncated Fourier reconstruction (first n = 18 harmonics) of photocurrent transients. **b** Spatial distribution of the average horizontal photocurrent measured directly using an electrometer. The scale bars are 500 µm.

Supplementary Fig. 15 shows the spatial distributions of the band-limited photocurrent I_{BL} (a) and the average photocurrent I_{AVG} (b) measured directly using a Keysight B2987B electrometer with a 10 s integration time. The average photocurrent measured using the electrometer with long integration times is close to zero, reflecting the absence of net charge transfer and confirming the purely displacement nature of the observed current. By contrast, the band-limited photocurrent yields a signal that provides a direct mapping of the local electrostatic potential distribution averaged over the illuminated volume of the sample in the horizontal direction.

Supplementary Note 9. Fitting the photocurrent decays to different models

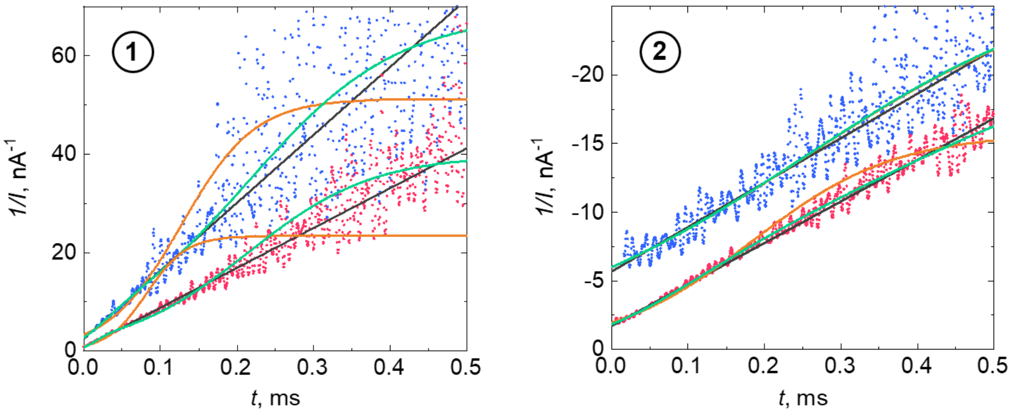

Supplementary Fig. 16. Photocurrent decays fitted to various models. Current decays measured in the horizontal direction on a sample isolated from stray light (blue dots) and under diffuse ambient illumination of 0.01 sun (pink dots) at the locations indicated in Fig. 4b plotted as $1/I(t)$ vs t. Solid curves represent fits to inverse time (black), exponential (orange), and bi-exponential (green) models.

Supplementary Fig. 16 shows the $I(t)$ data in regime II (Fig. 3g, the Discussion section of the main text) fitted to the inverse time model $I(t) \propto 1/(t + const)$, the exponential model $I(t) \propto \exp(-t/\tau)$, and the bi-exponential model $I(t) \propto \exp(-t/\tau_1) + \exp(-t/\tau_2)$. The inverse time model $I(t) \propto 1/(t + const)$ clearly provides the best fit to the experimental data.

Supplementary Note 10. Estimation of the characteristic charge density $\bar{n}$

The energy $E(\mathbf{k})$ of a massive quasiparticle inside a semiconductor barrier can be described by a Dirac-like equation

$$E^2(\mathbf{k}) = \pm\sqrt{\left(\frac{\Delta_{\mathrm{g}}}{2}\right)^2 + (\hbar V_0 \mathbf{k})^2}, \tag{10.1}$$

where Δ_g is the band gap energy, $\mathbf{k}$ is the wavevector, and V_0 is the potential within the barrier region. From this

$$E(\mathbf{k}) = \pm\sqrt{\left(\frac{\Delta_g}{2}\right)^2 + (\hbar V_0 \mathbf{k})^2} \approx \pm\left(\frac{\Delta_g}{2} + \Delta_g\left(\frac{\hbar V_0 \mathbf{k}}{\Delta_g}\right)^2\right). \quad (10.2)$$

For small $\mathbf{k}$, we can write

$$\Delta_g\left(\frac{\hbar V_0 \mathbf{k}}{\Delta_g}\right)^2 \equiv \frac{\hbar^2 \mathbf{k}^2}{2m^*}, \quad (10.3)$$

where m^* is the effective mass of a particle. The potential V_0 can now be expressed as

$$V_0^2 = \frac{\Delta_g}{2m^*}, \quad (10.4)$$

which when substituted into Eq. 10.1 gives us

$$E^2(\mathbf{k}) = \left(\frac{\Delta_g}{2}\right)^2 + \frac{\Delta_g \hbar^2 \mathbf{k}^2}{2m^*}. \quad (10.5)$$

If we define the characteristic quasi-classical momentum $\mathbf{k}_0$ inside the forbidden region as $E(\mathbf{k} = \mathbf{k}_0) = 0$, then

$$\mathbf{k}_0 = \pm i\sqrt{\left(\frac{\Delta_g}{2}\right)^2 \frac{2m^*}{\Delta_g \hbar^2}} = \pm i\sqrt{\frac{\Delta_g m^*}{2\hbar^2}}, \quad (10.6)$$

and the transmission coefficient through the barrier can be expressed as

$$T \sim \exp(-2|\mathbf{k}_0|d) = \exp(-2\kappa d), \quad (10.7)$$

where $|\mathbf{k}_0| \equiv \kappa$. The characteristic charge density can now be estimated by considering the decrease in the effective band gap energy Δ_g caused by electric field $\mathbf{E}$ resulting from the accumulation of charge carriers at the domain wall

$$\delta\Delta_g = -e\mathbf{E}d = -4\pi\sigma d e, \quad (10.8)$$

where e is the elementary charge, d is the domain wall thickness, and σ is the surface charge density at the domain wall. The tunneling rate R through the charged domain wall can be then expressed as

$$R \sim \exp(-2\kappa d) = \exp\left(-2\sqrt{\frac{2m^*(\Delta_g + \delta\Delta_g)}{\hbar^2}}\, d\right). \quad (10.9)$$

By expanding the expression for the decay constant κ and substituting $\delta\Delta_g$ with Eq. 10.8, we arrive at the modified tunneling rate equation

$$R \sim \exp(-2\kappa d) = \exp\left(\sqrt{\frac{2m^*}{\Delta_g \hbar^2}} 4\pi d^2 \sigma e\right). \tag{10.10}$$

At the same time, we found that the tunneling rate is exponentially sensitive to the carrier density n (see Eq. 2 in the main text)

$$R \sim \exp(n/\bar{n}) \equiv \exp(\sigma/\bar{\sigma})\,, \tag{10.11}$$

where $\bar{n}$ and $\bar{\sigma}$ are characteristic volume and surface charge densities, respectively. By combining Eq. 10.10 and Eq. 10.11, we arrive at the following expression for the characteristic surface charge density $\bar{\sigma}$

$$\bar{\sigma} = \sqrt{\frac{\Delta_g \hbar^2}{2m^*}} \frac{1}{4\pi d^2} \frac{1}{e} \approx 0.1 \text{ [}e \text{ per } a^2\text{]}. \tag{10.12}$$

Here we use the literature values for the band gap energy $\Delta_g \approx 2.3\ eV$ and carrier effective mass $m^* \approx 0.13$ [11] in $MAPbBr_3$ and assuming the thickness of the domain wall d is on the order of the lattice constant a, to obtain the value above. This is consistent with the values obtained from numerical simulations investigating the effects of charged domain walls on the electronic structure of $MAPbX_3$ lead-halide perovskites reported in [12].

Supplementary References

1. Collett, E. *Field Guide to Polarization*, vol. FG05 of Field Guides (SPIE Press, 2005).
2. Kawamura, Y., Mashiyama, H. & Hasebe, K. Structural study on cubic–tetragonal transition of $CH_3NH_3PbI_3$. *J. Phys. Soc. Jpn.* **71**, 1694–1697 (2002).
3. Mashiyama, H., Magome, E., Kawamura, Y. & Kubota, Y. Displacive character of the cubic-tetragonal transition in $CH_3NH_3PbX_3$. *J. Kor. Phys. Soc.* **42**, 1026–1029 (2003).
4. Bari, M., Bokov, A. A. & Ye, Z.-G. Ferroelastic domains and phase transitions in organic-inorganic hybrid perovskite $CH_3NH_3PbBr_3$. *J. Mater. Chem. C* **9**, 3096–3107 (2021).
5. Haynes, W. M., Ed., *CRC Handbook of Chemistry and Physics* (CRC Press, 95th ed., 2014).
6. Takahashi, T. Solid silver ion conductors. *J. Appl. Electrochem.* **3**, 79–90 (1973).
7. Lee, W. T., Salje, E. K. H. & Bismayer, U. Structure and transport properties of ferroelastic domain walls in a simple model. *Ph. Transit.* **76**, 81–102 (2003).
8. Cao, Y., Shen, J., Randall, C. & Chen, L.-Q. Effect of multi-domain structure on ionic transport, electrostatics, and current evolution in $BaTiO_3$ ferroelectric capacitor. *Acta Mater.* **112**, 224–230 (2016).
9. Sluka, T., Tagantsev, A. K., Bednyakov, P. & Setter, N. Free-electron gas at charged domain walls in insulating $BaTiO_3$. *Nat. Commun.* **4**, 1808 (2013).
10. Armistead, W. H. & Stookey, S. D. Photochromic silicate glasses sensitized by silver halides. *Science* **144**, 150–154 (1964).
11. Becker, M. A. et al. Bright triplet excitons in caesium lead halide perovskites. *Nature* **553**, 189–193 (2018).
12. Liu, S. et al. Ferroelectric domain wall induced band gap reduction and charge separation in organometal halide perovskites. *J. Phys. Chem. Lett.* **6**, 693–699 (2015).
