# Supplementary material for: Flexoelectric domain walls enable charge separation and transport in cubic perovskites
Source: Nat Commun. 2026 Feb 16;17:946. doi: 10.1038/s41467-026-68660-5 (PMC12909952; doi:10.1038/s41467-026-68660-5)
Supplement: Supplementary file 2 — Description of Additional Supplementary Files [file 41467_2026_68660_MOESM2_ESM.pdf]

Description of Additional Supplementary Files

File Name: Supplementary Movie 1
Description: $MAPbBr_3$ single crystal in a crossed polarizer setup.

File Name: Supplementary Movie 2
Description: Bright-field optical sectioning of dendritic structures formed in a $MAPbBr_3$ single crystal after application of an electric field.

File Name: Supplementary Movie 3
Description: 3D reconstruction of silver dendrites formed in a $MAPbBr_3$ single crystal after application of an electric field.
